# General practice organisational models and heart failure medication adherence: multi-level evidence from a regional cohort in Emilia-Romagna, Italy

**DOI:** 10.1093/intqhc/mzaf124

**Published:** 2025-12-19

**Authors:** Jacopo Palombarini, Simona Rosa, Davide Golinelli, Vera Maria Avaldi, Giorgia Vallicelli, Michela Fantini, Maria Pia Fantini, Jacopo Lenzi

**Affiliations:** Department of Biomedical and Neuromotor Sciences, University of Bologna, Bologna, 40126, Italy; Department of Biomedical and Neuromotor Sciences, University of Bologna, Bologna, 40126, Italy; Department of Life Sciences, Health, and Health Professions, Link Campus University, Rome, 00165, Italy; Unit of Health Services Research, Evaluation and Policy, Local Health Authority of Romagna, Ravenna, 48121, Italy; Unit for Monitoring of Care Pathways and Clinical Processes, Local Health Authority of Bologna, Bologna, 40124, Italy; Unit of Health Services Research, Evaluation and Policy, Local Health Authority of Romagna, Ravenna, 48121, Italy; Unit of Health Services Research, Evaluation and Policy, Local Health Authority of Romagna, Ravenna, 48121, Italy; Alma Mater Professor, University of Bologna, Bologna, 40126, Italy; Department of Biomedical and Neuromotor Sciences, University of Bologna, Bologna, 40126, Italy

**Keywords:** primary care/general practice, cardiovascular diseases, elderly, care pathways/disease management, quality indicators, case-mix or risk adjustment, health policy, statistical methods, improvement science

## Abstract

**Background:**

Medication adherence is essential for improving heart failure outcomes yet remains suboptimal. Organisational models in general practice—such as group practices and Community Health Centres—have long been promoted as a means to strengthen primary care and are currently undergoing national reform. However, their impact on adherence remains unclear. This study investigated whether general practice organisational arrangements were associated with adherence to therapies recommended by clinical guidelines for heart failure.

**Methods:**

We conducted a retrospective cohort study using linked administrative data from the Romagna Local Health Authority (Northern Italy), encompassing all adults discharged with an incident diagnosis of heart failure between January 2020 and March 2023. The primary outcome was adherence to angiotensin-converting enzyme inhibitors, angiotensin II receptor blockers, or β-blockers over one year, based on pharmacy claims. The exposure was the general practice organisational model: group practice within a Community Health Centre, group practice outside a Community Health Centre, or solo/networked practice. We used multilevel logistic regression to estimate adjusted associations, accounting for patient- and context-level confounders, with additional stratified analyses by health district.

**Results:**

No systematic association emerged between general practice organisational models and adherence in the overall cohort of 3304 patients with heart failure. However, in one district (Rubicone), group practices were associated with higher adherence (angiotensin-converting enzyme inhibitors/angiotensin II receptor blockers: odds ratio = 3.00, 95% confidence interval 1.48–6.09, *P *= .002; β-blockers: odds ratio = 1.81, 95% confidence interval 0.98–3.37, *P *= .06). Residual variation between general practitioners was modest but not negligible.

**Conclusion:**

Organisational arrangements alone may be insufficient to improve adherence in heart failure care. Their effectiveness likely depends on how they are implemented and supported at the local level, through clinical leadership, specialist involvement, and integration across care settings. As new national reforms promote broader structural change, our findings underscore the importance of local facilitators and context-sensitive implementation. These insights are particularly relevant for understanding the operational strengths and weaknesses of legacy models that are now being phased out.

## 1. Introduction

Non-adherence to long-term therapies affects up to 50% of patients in high-income countries [1]. This contributes to avoidable hospitalisations, mortality, and healthcare costs [2]. As highlighted by literature reviews, adherence is shaped not only by patient characteristics and treatment complexity but also by organisational and systemic factors [3], including patient beliefs and preferences [4].

In response to rising multimorbidity and ageing, many countries have sought to strengthen primary care and redefine the role of general practitioners (GPs). In Italy, GPs are the main access point to care and act as gatekeepers to specialist services. Their practice is governed by national, regional, and local ­agreements that have promoted new collaborative ­models [5–7].

The 2000 National Collective Agreement (*Accordo collettivo nazionale—*ACN) introduced three GP association types: simple (‘solo’) associations (common diagnostic and therapeutic guidelines), networked practices (shared electronic records, not co-located), and group practices (co-location of GPs and staff). Studies on these models have shown mixed results, suggesting that their effectiveness may depend on implementation and local context [8–10]. More recently, research has focused on how organisational features, including teamwork and care coordination, affect medication adherence [11, 12].

In parallel, many systems have promoted integrated, community-based approaches to manage complex chronic patients [2, 13]. Emilia-Romagna has pioneered this direction, establishing over 120 *Case della comunità* (Community Health Centres—CHCs) that host multi-professional teams and aim to foster integration between primary care, social assistance, and preventive services. However, GP participation in CHCs remains optional, and most associations still operate outside them.

In Italy, this organisational landscape is now changing: the 2024 ACN and ongoing regional agreements aim to replace legacy models with *Aggregazioni funzionali territoriali* (AFTs) and *Unità complesse di cure primarie* (UCCPs), emphasising multi-professional governance and structured population health management. Understanding how previous configurations functioned—before their transformation—can inform these reforms and help preserve elements that worked well in practice.

Among chronic diseases, heart failure (HF) poses a major public health burden, particularly in older adults [14]. Adherence to evidence-based HF therapies improves outcomes and reduces hospitalisations [2, 15]. Yet the role of GP organisational models in supporting such adherence remains underexplored.

This study addresses this gap using data from a large HF cohort in Romagna, a sub-region of Emilia-Romagna. We examine whether different GP arrangements—as defined by the 2000 ACN—were associated with adherence to recommended therapies. We hypothesised that configurations fostering structured collaboration and proximity to other professionals could support better adherence. Clarifying these associations may offer timely insights as Italy transitions toward more integrated, multi-professional models of care.

## 2. Methods

### 2.1 Study design and data sources

We conducted a retrospective cohort analysis to evaluate the association between general practice organisational models and pharmacological adherence in HF patients, adjusting for patient- and context-level confounders.

The study was set in the Romagna Local Health Authority (*AUSL Romagna*), a large subregional health system in Emilia-Romagna, Northern Italy, serving nearly 1.1 million residents and encompassing over 800 GPs.

The study adhered to the *REporting of studies Conducted using Observational Routinely-collected health Data* (RECORD) guidelines (see Supplementary Data) and relied on an integrated database built through deterministic record linkage via unique patient identifiers, drawing from the following administrative sources listed in Box 1.

Box 1. Data sources used for deterministic record linkage

Hospital discharge records (*Schede di dimissione ospedaliera—*SDO), coded using the International Classification of Diseases, Ninth Revision, Clinical Modification (ICD-9-CM), containing diagnostic and procedural information.

Pharmacy claims, including drugs reimbursed by the Italian National Health Service and dispensed by hospital pharmacies (*Farmaci a erogazione diretta—*FED) or community pharmacies following GP/specialist prescriptions (*Assistenza farmaceutica territoriale—*AFT), coded using the Anatomical Therapeutic Chemical (ATC) classification.

Records on residential and semi-residential elderly care (*Assistenza residenziale e semi-residenziale anziani—*FAR).

Integrated home care services (*Assistenza domiciliare integrata—*ADI).

Specialist ambulatory care (*Assistenza specialistica ambulatoriale—*ASA), including outpatient visits, diagnostics, and laboratory tests from public or accredited providers.

Vital registration data (*Registro mortalità* – REM).

GP administrative registry, reporting physician identifiers, practice setting, organisational model, and CHC co-location.

As the linkage process was entirely deterministic and involved no exclusions, we did not include a flow diagram.

### 2.2 Study population

We included all adults residing in AUSL Romagna discharged from any Italian hospital with a diagnosis of HF between 1 January 2020 and 31 March 2023. Patients were identified from SDO records using a validated algorithm based on ICD-9-CM codes in the primary diagnostic position (source: https://pne.agenas.it/): 428.x (HF), 402.x1 (hypertensive heart disease with HF), 404.x1 and 404.x3 (hypertensive heart and renal disease with HF), and 398.91 (rheumatic HF).

Re-hospitalisations within two days were merged into single episodes. The index date was the discharge date of the index episode. Patients were followed for up to 365 days or until death or admission to long-term residential care. Only the first eligible discharge was retained in case of multiple hospitalisations.

Exclusion criteria—some based on [16]—are listed in Box 2.

Box 2. Exclusion criteria applied to the initial cohort

Any prior hospitalisation with a primary/secondary HF diagnosis, or DRG 127 (“HF and shock”) within a six-year lookback, to identify incident cases.

Residence in a long-term care facility (FAR) at discharge, as pharmacy claims are unavailable.

Hospital stays >95th percentile (>30 days), likely reflecting unstable or complex conditions.

Age <5th percentile (<60 years), indicating atypical therapeutic patterns.

Death within 30 days post-discharge.

Hospitalisation for >30% of follow-up, due to unobservable pharmacy claims.

Absence of GP registration in AUSL Romagna.

Unstable or missing GP organisational model or setting.

The final study population was derived from 6,057 eligible records, already partially filtered by the data custodian. Sample size details are reported in the Results.

### 2.3 Exposure variables

The main exposure was the GP’s organisational model, recorded in the AUSL Romagna administrative registry and based on the 2000 ACN typology. GPs were initially classified as follows: solo practice (outside shared facilities or team-based settings), networked practice (shared electronic records without formal/logistical integration), and group practice (GPs co-located or jointly operating in common facilities).

We also recorded whether GPs operated within CHCs, which implies multi-professional co-location and structured collaboration. Since only grouped GPs may work in CHCs, the two variables were combined. Furthermore, solo and networked practices were merged due to small numbers and service similarities. The final exposure categories were therefore: solo or networked GP practice; group practice outside a CHC; and group practice inside a CHC.

Exposure was assigned based on the GP covering >30 consecutive days during the first three months post-discharge. If two GPs met this, the first was assigned. If the GP changed model during follow-up, only the initial arrangement was retained.

### 2.4 Pharmacological outcomes

Adherence was assessed using pharmacy claims (AFT/FED), with drugs classified by ATC codes. We focused on two HF therapies endorsed by guidelines [14]: angiotensin-converting-­enzyme inhibitors (ACEIs) and angiotensin II receptor blockers (ARBs) (ATC C09), and β-blockers (ATC C07). These are used by the Italian Ministry of Health to monitor HF care. Although adherence indicators do not distinguish clinical subtypes, they are accepted proxies of therapeutic quality in administrative data [17].

Drugs were cumulated using Defined Daily Doses (DDDs). Adherence was defined as the Proportion of Days Covered (PDC), that is, proportion of days with drug availability. Overlapping prescriptions were accounted for; early refills were excluded. A PDC threshold of 75% was used to classify patients as adherent (PDC ≥75%) or non-/partially adherent (PDC <75%) [18]. Patients without prescriptions were considered non-adherent. A sensitivity analysis using the Medication Possession Ratio confirmed the robustness of results and did not alter the conclusions (data not shown).

We did not examine combination therapy due to missing clinical details and misinterpretation risks. As an alternative, we restricted the cohort to patients with ≥1 filled prescription in the first trimester, as a proxy for persistence. Sensitivity results remained unchanged (data not shown).

### 2.5 Potential confounders

Covariates were selected *a priori* based on the literature and data availability. At patient level: sex, age, length of stay, Modified-Chronic Disease Score (M-CDS) based on prior 2-year prescriptions [19], and binary indicators of prior adherence to ACEIs/ARBs and β-blockers over six months.

We also included a post-discharge cardiology visit within 90 days (a known adherence predictor) [20], as well as early rehospitalisation and domiciliary care intensity (absent/low/medium/high) during the same period, as proxies of complexity.

At GP level, we considered sex, age, and patient roster size. Continuous variables were left uncategorised in boosting; quantile-based categorical versions based on quantile splits (e.g. age groups) were used in the lasso selection (see Supplementary Data).

The GP’s district of practice was included as a potential confounder and effect modifier; hospital of discharge was excluded due to strong collinearity.

### 2.6 Statistical analysis

Numerical variables were summarised as means and standard deviations (SDs); categorical variables as counts and percentages.

We investigated the association between general practice organisational models and adherence to pharmacological therapy using multi-level mixed-effects logistic regression models—one per drug class—to account for the hierarchical data structure (patients nested within GPs).

Details on model construction and techniques used to reduce confounding and strengthen causal inference are provided in the Supplementary Data. Fixed-effects estimates are reported as odds ratios (ORs) with 95% confidence intervals (CIs). Random effects (intercepts) are summarised as variance components on the logit scale and as variance partition coefficients (VPCs), indicating the proportion of residual variance attributable to between-GP differences.

### 2.7 Stratified analysis by health district

In Italy’s decentralised healthcare system, health districts oversee primary care delivery and coordinate access to outpatient and inpatient services, including diagnostics and specialist care. Despite efforts by local health authorities to ensure equity, substantial variability may persist across districts.

To account for this heterogeneity, we tested statistical interactions between the GP’s district and the exposure (i.e. general practice model), operationalised as a binary indicator (solo/networked vs. group-based care). The rationale for this dichotomisation, along with further analytical details, is provided in the Supplementary Data.

All analyses were performed using Stata 18, R 4.3.3, and Python 3.11. The significance level was set at 0.05; all statistical tests were two-sided.

## 3. Results

**Figure 1.**
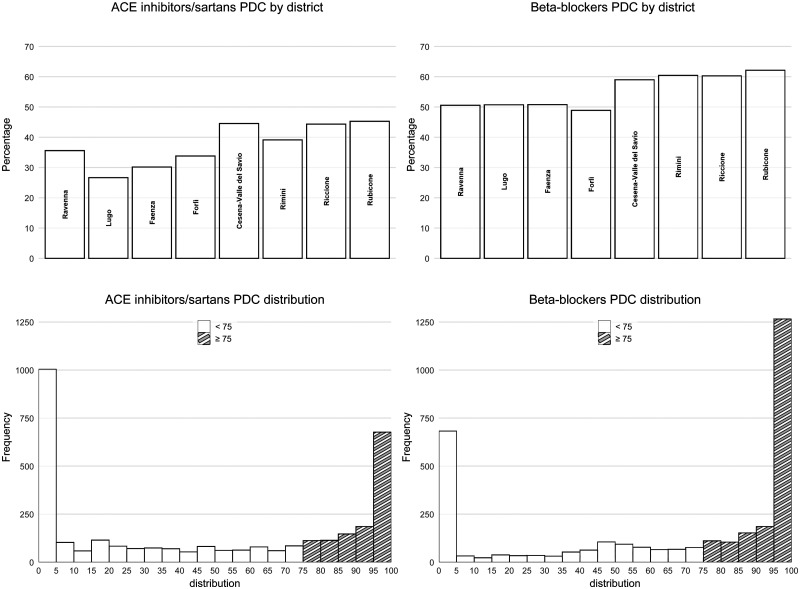
Distribution of ACEIs/ARBs and β-blockers PDC, overall and by GP’s district of practice. Abbreviations: ACEIs, ACE Inhibitors; ARBs, Angiotensin II Receptor Blockers (sartans); PDC, Proportion of Days Covered.

A total of 3304 HF patients, 697 GPs, 33 CHCs, and 118 discharging hospitals were included (Table 1). These patients were identified from 6057 eligible hospital discharges already partially filtered by the data custodian, yielding a final cohort inclusion rate of 54.5%. Mean patient age was 82.1 years (SD 8.6); 1743 (52.8%) were female. Over a median follow-up of 323 days, 651 patients (19.7%) died within one year of hospital discharge. Adherence rates (PDC ≥ 75%) were 37.4% (1237/3304) for ACEIs/ARBs and 55.1% (1821/3304) for β-blockers. Figure 1 displays PDC distributions by drug class and district.

**Table 1.** Data summary by GP’s district of practice

Districts	Patients	GPs	CHCs	Hospitals
Ravenna	621	122	7	19
Lugo	270	56	8	14
Faenza	252	58	5	13
Forlì	544	110	5	17
Cesena-Valle del Savio	339	77	1	10
Rimini	690	143	2	20
Riccione	345	77	2	11
Rubicone	243	54	3	14
*Total*	*3304*	*697*	*33*	*118*

Abbreviations: CHC, Community Health Centre; GP, General Practitioner.

Among the 697 GPs, 295 (42.3%) were female; the mean age was 57.2 years (SD 10.6). In terms of organisational models, 999 patients (30.2%) were followed by GPs in solo or networked practice, 1590 (48.1%) by group practices outside CHCs, and 715 (21.6%) by group practices within CHCs. Overall, 156 GPs (22.4%) worked in a CHC, 325 (46.6%) worked in groups outside CHCs, 141 (20.2%) were part of network practices, and 75 (10.8%) worked as ‘solo’ professionals.

### 3.1 Multi-level analysis

After weighting and doubly robust adjustment, no significant association emerged between GP organisational models and adherence in the overall population (Table 2). Compared to solo or networked practices:

**Table 2.** Results of multilevel logistic regression models with inverse probability of treatment weighting (IPTW) and doubly robust adjustment

	ACEIs/ARBs adherence			β-blockers adherence		
	OR	95% CI	*P*	OR	95% CI	*P*
Age group, y						
≤75	Ref.			Ref.		
76–85	0.56	0.46–0.69	<.001	0.57	0.46–0.71	<.001
86–90	0.41	0.32–0.51	<.001	0.44	0.34–0.57	<.001
>90	0.51	0.39–0.66	<.001	0.33	0.25–0.44	<.001
Female sex	1.01	0.85–1.20	.91	1.20	1.01–1.41	.034
Prior adherence to same drug class (PDC ≥75%)	5.03	4.12–6.15	<.001	5.49	4.66–6.46	<.001
Cardiology visit within 90 days of discharge	1.06	0.87–1.30	.54	1.37	1.11–1.69	.004
Home care intensity in first 90 days						
None	Ref.			Ref.		
Low	0.51	0.41–0.64	<.001	0.80	0.65–1.00	.047
Medium	0.50	0.38–0.65	<.001	0.81	0.63–1.05	.11
High	0.38	0.23–0.64	<.001	0.82	0.51–1.32	.42
GP’s district of practice						
Ravenna						
Lugo	0.59	0.41–0.86	.005	0.92	0.63–1.34	.67
Faenza	0.75	0.53–1.05	.09	0.92	0.64–1.32	.65
Forlì	0.91	0.69–1.22	.54	0.80	0.60–1.07	.13
Cesena-Valle del Savio	1.47	1.05–2.05	.025	1.23	0.90–1.67	.19
Rimini	1.20	0.92–1.56	.18	1.38	1.05–1.81	.022
Riccione	1.39	1.00–1.94	.053	1.35	0.97–1.86	.07
Rubicone	1.51	1.04–2.20	.030	1.40	1.01–1.96	.045
Type of GP practice						
Solo or networked						
Group outside CHC	0.96	0.79–1.17	.70	0.93	0.76–1.13	.45
Group inside CHC	1.07	0.84–1.36	.59	0.91	0.72–1.16	.44

Abbreviations: ACEIs, ACE Inhibitors; ARBs, Angiotensin II Receptor Blockers; CI, Confidence Interval; CHC, Community Health Centre; GP, General Practitioner; OR, Odds Ratio; PDC, Proportion of Days Covered.

- Group practices outside CHCs showed no significant association (ACEIs/ARBs: OR=0.96, 95% CI 0.79–1.17, *P*=.70; β-blockers: OR=0.93, 95% CI 0.76–1.13, *P*=.45).
- Group practices within CHCs also showed no association (ACEIs/ARBs: OR=1.07, 95% CI 0.84–1.36, *P*=.59; β-blockers: OR=0.91, 95% CI 0.72–1.16, *P*=.45).

GP-level random intercepts showed limited variability after adjustment, with variance estimates of 0.190 (95% CI 0.097–0.372) for ACEIs/ARBs and 0.182 (95% CI 0.083–0.398) for β-blockers. This corresponds to VPCs of 5.5% and 5.2%, respectively, indicating the proportion of total residual variance in adherence attributable to differences between GPs.

To assess potential effect modification by local context, interaction terms between district and GP model (group vs solo/networked) were considered. A significant interaction was found for the Rubicone district (*P *= .003 for ACEIs/ARBs; *P *= .017 for β-blockers). In all other districts, interaction *P*-values ranged between .11 and .95 for ACEIs/ARBs, and between .08 and .70 for β-blockers.

Stratified estimates were therefore reported exclusively for Rubicone. Here, patients followed by GPs in group practices—regardless of whether inside or outside CHCs—had significantly higher odds of adherence compared to those in solo or networked practices. For ACEIs/ARBs, the odds ratio was 3.00 (95% CI 1.48–6.09; *P *= .002), indicating a strong association. For β-blockers, the association was weaker and of borderline significance (OR = 1.81, 95% CI 0.98–3.37; *P *= .06), suggestive of a trend.

## 4. Discussion

### 4.1 Statement of principal findings

This study investigated whether organisational models in general practice—defined by the 2000 ACN and including co-location within CHCs—were associated with medication adherence among HF patients in Romagna. Despite adjustment for individual and contextual factors, no systematic association emerged between group practice models and adherence to ACEIs/ARBs or β-blockers. Most patients received care outside CHCs, yet this did not appear to affect their likelihood of receiving long-term therapy. These results suggest that structural arrangements alone may not suffice to improve adherence in complex chronic conditions.

However, stratified analyses revealed a notable exception: in the Rubicone district, group practices—regardless of CHC location—were associated with significantly higher adherence. This localised effect contrasts with the overall null results and highlights the importance of context-specific factors. Possible contributors may include stronger clinical leadership, a more cohesive team climate, better communication, or clearer role distribution—elements known to influence quality of care in primary care settings [21]. These findings align with the modest but non-negligible between-GP variation observed in our models, suggesting that micro-level dynamics and informal routines may help shape adherence.

### 4.2 Strengths and limitations

The study’s strengths include the use of a large, population-based cohort and multilevel modelling to account for clustering. This enabled meaningful district-level analyses and detection of local outliers.

Several limitations are worth noting. First, the observational design precludes causal inference despite advanced adjustment. Second, exposure classification relied on administrative data, which may not reflect actual implementation. Third, adherence was based on dispensed claims and WHO DDDs, potentially underestimating true use in HF. Fourth, clinical variables (e.g. disease severity, contraindications) were unavailable, possibly introducing residual confounding. In addition, we did not assess clinical outcomes. This was partly due to data constraints but also reflects conceptual challenges. Rehospitalisations, for instance, are difficult to interpret, and organisational exposures tend to exert indirect and attenuated effects on distal, hard outcomes. These effects are typically mediated through numerous care processes, which would require larger samples to assess causal pathways. Fifth, generalisability may be limited to systems with similar GP infrastructure. Sixth, the Rubicone effect lacks qualitative corroboration: stakeholder interviews or positive deviance analysis could help identify enabling mechanisms—such as GP seniority or previous hospital experience—not captured in our data [22]. Similarly, informal relationships—whether between GPs and their patients or among healthcare professionals—may also shape adherence behaviours but remain unobservable in administrative data and thus could not be assessed in this study. Seventh, we lacked historical data on GP availability (i.e. office hours), which could influence access and adherence. Finally, the study period overlapped with COVID-19, whose impact on care and adherence remains uncertain.

### 4.3 Interpretation within the context of the wider literature

Our findings are reinforced by prior evidence on health system reforms [23], which emphasises the importance of adaptive implementation, stakeholder engagement, and distributed leadership. In this view, it is not the legal or structural form of GP practice that matters—especially in complex conditions such as HF—but how it functions in practice. Our results resonate with this perspective: configurations enabling structured collaboration and proximity may support better adherence—but only when enacted effectively within a supportive context.

Importantly, co-location within CHCs—as defined in administrative data—does not guarantee integrated, multi-professional care. Physical proximity to other professionals does not imply shared governance, nurse-led interventions, or collaborative workflows. Without implementation indicators (e.g. joint care plans, team meetings, care pathways), it is unclear whether CHC-based models operated as intended. Conversely, some GPs outside CHCs may have achieved functional integration through alternative means, such as informal networks or shared protocols. This ambiguity cautions against equating structural location with actual practice transformation.

Indeed, access to community nursing, specialist support, and proactive care coordination—often orchestrated at district level—may matter more for adherence than practice setting alone. This echoes empirical findings showing that team dynamics and clinical governance better predict care quality than aggregation type [9]. International evidence reinforces this view [24, 25]: without genuine integration and operational leadership, structural reforms rarely improve outcomes. Zwiep et al. similarly show that group practices vary widely in provider mix, governance, and internal functioning—underscoring that effectiveness stems from enacted collaboration, not legal structure. Relational processes—such as communication and shared norms—may account for up to one-quarter of team effectiveness [26].

Although our models did not include hospital-level random effects, one post-discharge feature—early cardiology follow-up—emerged as a significant predictor of β-blocker adherence. This finding aligns with prior research showing that timely specialist involvement improves therapeutic continuity and reduces rehospitalisations [27–30]. Italian studies confirm this association, highlighting the role of cardiology engagement in supporting adherence to evidence-based therapies [20]. These results underscore the importance of structured discharge planning and integrated pathways between hospital and community.

### 4.4 Implications for policy, practice and research

The organisational models examined in this study—GP associations and their co-location within CHCs—reflect the framework established by the 2000 ACN. These legacy configurations were based on the prevailing assumption that mono-professional GP models could still address the growing burden of chronic diseases. However, the shift towards AFTs and UCCPs marks a structural reorientation towards integrated, multi-professional governance.

Although the models analysed remained widespread during the study period, they are now being phased out or reshaped under recent national and regional reforms. Our findings should therefore not be interpreted as an endorsement of outdated structures, but rather as an opportunity to understand which operational features may have contributed to—or hindered—their effectiveness.

This knowledge can inform the ongoing implementation of new organisational models. Specifically, future policy should prioritise the enabling conditions that support collaboration in practice—not just on paper. For researchers, these findings point to the need for deeper qualitative and mixed-methods investigations into micro-level team dynamics, leadership patterns, and the informal processes that drive effective primary care.

## 5. Conclusions

This study highlights the limitations of relying solely on formal organisational models—such as GP associations or co-location within CHCs—to improve medication adherence in HF care. Structural arrangements, even when endorsed by national frameworks, may be insufficient without enabling local conditions and effective implementation.

Our findings suggest that operational mechanisms—such as clinical leadership, team-based workflows, and shared governance—are likely more consequential than legal form alone. The significant adherence advantage observed in one district underscores the importance of context, micro-level dynamics, and informal collaboration in shaping outcomes.

Policy efforts should therefore move beyond structural prescriptions and focus on fostering organisational environments that function effectively in practice. Investing in integrated care pathways, interprofessional coordination, and context-sensitive implementation strategies may offer more promising avenues for improving adherence in chronic care.

Finally, the predictive role of early cardiology follow-up should not be seen in isolation. Rather, it illustrates how targeted, cross-setting interventions—linking hospital and primary care—can enhance therapeutic continuity and complement broader reforms.

## Supplementary Material

mzaf124_Supplementary_Data

## Data Availability

Data and scripts are available upon reasonable request. Requests to access the datasets should be directed to the corresponding author.
