# Supplementary material for: General practice organisational models and heart failure medication adherence: multi-level evidence from a regional cohort in Emilia-Romagna, Italy
Source: Int J Qual Health Care. 2025 Dec 19;38(1):mzaf124. doi: 10.1093/intqhc/mzaf124 (PMC12933156; doi:10.1093/intqhc/mzaf124)
Supplement: mzaf124_Supplementary_Data [file mzaf124_supplementary_data.zip › suppl_2025.07.17.docx]

**SUPPLEMENTARY INFORMATION ON STATISTICAL ANALYSES**

**Propensity score estimation**

To reduce confounding bias and strengthen causal inference, we applied inverse probability of treatment weighting (IPTW) based on general-practice propensity scores. These scores represent the probability of receiving a given organisational model of care and were estimated using Multiple Additive Regression Trees (MART) with a gradient boosting algorithm, incorporating all pre-specified potential confounders [1]. The boosting procedure used a maximum tree depth of 5, up to 20,000 iterations, 50% bagging, and a shrinkage factor of 0.01. Given the multi-valued exposure (solo or networked practice; group practice outside Community Health Centres; group practice within Community Health Centres), a multinomial distribution was assumed for propensity score estimation.

Because our estimand of interest was the average treatment effect in the population, each observation was weighted by the inverse probability of receiving the “treatment” actually received. Propensity scores were truncated at the 99^th^ percentile for all models to limit extreme values and preserve covariate balance.

**Doubly robust estimation and covariate selection**

To further mitigate residual confounding after the boosting stage, we adopted a doubly robust estimation approach, which entails including in the outcome model the covariates already used in the propensity score estimation [2,3]. However, to improve estimate efficiency and avoid overfitting, covariates were pre-selected using a preliminary double-selection lasso logistic regression with clustered standard errors, except for patient sex and age, which were forced into the model [4].

**Model estimation and random effects**

Final mixed-effects models were fitted using mean–variance adaptive Gauss–Hermite quadrature, a method shown to improve estimation accuracy in multi-level modelling [5]. The likelihood ratio test did not support the inclusion of random slopes for any patient-level covariates; therefore, all covariates were modelled as fixed effects. The variance partition coefficients (VPCs), indicating the proportion of residual variance attributable to differences across general practitioners (i.e., across random intercepts), were calculated following Snijders and Bosker’s classical approach.[6]

**Interaction and stratified analysis**

We tested statistical interactions between the general practitioner’s district of practice and the exposure variable (i.e., general practice model), operationalised as a binary indicator contrasting solo or networked practices versus group-based care. This simplification was adopted to address the pronounced imbalance in the local distribution of group practices located inside versus outside Community Health Centres: in several districts, one of the two categories was underrepresented, leading to unstable estimates and interpretative challenges. Although structural integration may differ between the two settings, they often share similar care delivery patterns at the local level—particularly in smaller districts. Therefore, for the purpose of district-level comparisons, the analysis focused on the broader contrast between solo/network and group-based practice.

Stratified analyses were then performed only for districts showing a statistically significant interaction with the binary exposure. The model specification remained unchanged, and weights and covariates were retained from the overall Romagna cohort.

**References**

[1] McCaffrey DF, Griffin BA, Almirall D, Slaughter ME, Ramchand R, Burgette LF. A tutorial on propensity score estimation for multiple treatments using generalized boosted models. Stat Med. 2013 Aug 30;32(19):3388-414. doi: 10.1002/sim.5753. Epub 2013 Mar 18. PMID: 23508673; PMCID: PMC3710547.

[2] Austin PC, Stuart EA. Moving towards best practice when using inverse probability of treatment weighting (IPTW) using the propensity score to estimate causal treatment effects in observational studies. Stat Med. 2015 Dec 10;34(28):3661-79. doi: 10.1002/sim.6607. Epub 2015 Aug 3. PMID: 26238958; PMCID: PMC4626409.

[3] Hashimoto Y, Yasunaga H. Theory and practice of propensity score analysis. Ann Clin Epidemiol. 2022 Oct 3;4(4):101-109. doi: 10.37737/ace.22013. PMID: 38505253; PMCID: PMC10760486.

[4] Belloni A, Chernozhukov V, Wei Y. Post-selection inference for generalized linear models with many controls. J Bus Econ Stat. 2016;34(4):606–619. doi: 10.1080/07350015.2016.1166116.

[5] Pinheiro JC, Chao EC. Efficient Laplacian and adaptive Gaussian quadrature algorithms for multilevel generalized linear mixed models. J Comput Graph Stat. 2006;15(1):58–81. doi: 10.1198/106186006X96962.

[6] Snijders TAB, Bosker RJ. Multilevel Analysis: An Introduction to Basic and Advanced Multilevel Modeling. 2^nd^ ed. Los Angeles: Sage; 2012.
